# On Toxic Materials Released from the Fæces by Enemata and Poisoning the System

**Published:** 1898-09-03

**Authors:** James Oliver

**Affiliations:** Physician to the Hospital for Women, London


### MEDICAL PROGRESS AND HOSPITAL CLINICS.

[*The Editor will be glad to receive offers of co-operation and contributions from members of the profession. All letters should be addressed to* The Editor, at the Office, 28 & 29, Southampton Street, Strand, London, W.C.]

### ON TOXIC MATERIALS RELEASED FROM THE FÆCES BY ENEMATA AND POISONING THE SYSTEM.

By James Oliver, M.D., F.R.S.(Edin.), F.L.S., Physician to the Hospital for Women, London.

Under ordinary circumstances, although the fæces contain very powerful poisons, these are seldom absorbed to such an extent as to cause alarming symptoms, yet the introduction of soapy water into the lower bowel for the purpose of exciting purgation serves occasionally to set free some of these toxic materials, which are thereafter rapidly absorbed, and tend to produce symptoms of a more or less alarming and even dangerous character. In these cases the fæces, prior to the introduction of the soapy water with which they are brought into contact, appear to exist in a normal state, as no symptoms indicative of the absorption of deleterious materials from the intestinal canal are complained of until the enema is given.

Considering the very varied character of the food stuffs ingested, the many forms of lower organisms introduced with these, and the different digestive fluids thrown into the alimentary canal, it is not altogether astonishing that the introduction of fluids into the lower bowel, which may react chemically and otherwise upon the matter lodged therein, should occasionally result in the production of symptoms which are attributable to the release and absorption of poisonous agents. In consequence of the presence of lactic acid, which is developed from the carbo-hydrates ingested and other acids which are produced by putrefaction, the reaction of the contents of the rectum is often acid; sometimes, however, ammonia is present in such a quantity that the reaction is neutral or even alkaline. The nature of the reaction is, as we shall presently find, a question of some importance, as the chemical changes which will take place when an alkaline liquid like soapy water is thrown into the bowel will vary according as the intestinal contents are acid or alkaline. In commerce we have two varieties of soap—the hard and the soft—and as either may be used to make the so-called soap enema, and both are not equally powerful in setting free the poisonous materials locked up in the fæces, it is imperative that we should be familiar with the composition of each. Soaps are made by saponifying tallow, olive oil, and other fats with the caustic alkali soda or potash, and a solution of any of them is invariably alkaline. Those which contain soda constitute the hard soaps, and those which contain potash constitute the soft varieties; the latter contain always a very considerable excess of alkali, and are consequently very decidedly alkaline.

Ptomaines and leucomaines are found in and can be extracted from healthy fæces, and these are formed by the decomposition of proteid substances—such as albumen, fibrine, and gelatine—in the intestines. Some of these products of decomposition are poisonous, whilst others are not, and amongst those which are poisonous some are much more actively so than others. The poisonous properties of the ptomaines vary according to the extent to which the putrefactive process has proceeded. Some ptomaines are similar to the vegetable alkaloids, and most of those which have been obtained by the decomposition of albumen resemble in their action either muscarine or atropine. Those which resemble muscarine depress the circulation, and tend to cause diarrhœa, whilst those resembling atropine accelerate the pulse, and may produce an erythematous rash, which is very similar to that of scarlet fever. So long, however, as the ptomaines and leucomaines maintain the state in which they usually exist, they produce no constitutional disturbance; they exert their deleterious influences only when their combination becomes deranged. Although the toxicity of fæces is partly dependent upon ptomaines and leucomaines, it is chiefly due to the presence of salts of potash and ammonia. When potash salts are introduced immediately into the blood they are extremely toxic, and, except in the case of the cyanide salts, where the action is due to the acid, the effect is always independent of the acid, and is solely attributable to the base. Many of the alarming symptoms which occur in consequence of the administration of a soap enema are due to the rapid absorption of some salts of potass, as constitutional disturbances are much less likely to be engendered when the enema is made by means of soft instead of hard soap. The toxicity of fæces is also due to materials derived from the bile. When bile salts are injected into the blood, they cause slowing of the pulse and diminution of the body temperature. From the alvine dejections, therefore, we can obtain substances which not only slacken but accelerate the circulation, and substances which not only increase but decrease the body temperature. Some of the materials elaborated in the alimentary canal are evidently antagonistic to, and may tend to counteract, each other.

In cases of acute auto-infection from the lower bowel the body becomes covered with an erythematous rash akin to that of scarlet fever, and here and there petechiæ, the result of small hæmorrhages into the skin, are occasionally observed; the body temperature is elevated, and may show a rise of 4 deg. to 5 deg. F. above normal; the circulation is accelerated, and the pulse may number 110 to 120 per minute, and sometimes more or less abdominal pain is complained of. When these disturbances occur they are almost invariably induced by throwing a solution of hard or soda soap into the lower bowel; they very seldom follow the use of an enema of plain water, or one made with a potash or soft soap. It is highly probable, therefore, that the toxic symptoms thus produced are due not merely to the formation of an aqueous solution of some of the fæcal materials, but to some chemical change taking place between one or some of them and the alkali introduced by the enema. The alkali which is more likely to effect this change is soda and not potash, and we are somewhat justified in believing that the symptoms themselves are to a certain extent dependent upon the liberation and absorption of some of the potash, which, as I have already stated, is one of the chief toxic ingredients of the fæces. When the potash is thus chemically released, some ptomaines and leucomaines will almost invariably be set free at the same time, and the presence of these will modify the symptoms which might otherwise be attributed to the salts of potash alone. Vomiting is a symptom which is occasionally noted in association with this form of auto-infection, but there is seldom, if ever, evidence of disturbance of the glandular organs. When an enema of soapy water is given during convalescence from an acute disease, after parturition, or after some serious operation upon the abdominal or pelvic organs, and some or all of the constitutional disturbances which I have referred to are thereafter developed, it may be difficult to thoroughly appreciate their significance.

